# Mitral Valve Aneurysm With Severe Mitral Regurgitation and Pseudoaneurysm Formation: A Rare Complication of Infective Endocarditis

**DOI:** 10.7759/cureus.82491

**Published:** 2025-04-18

**Authors:** Kesar Prajapati, FNU Samaksh, Poornima Jaiswal Charpuria, Nisarg Desai, Ankit V Shah

**Affiliations:** 1 Internal Medicine, New York Medical College, Metropolitan Hospital Center, New York, USA; 2 Cardiology, U. N. Mehta Institute of Cardiology and Research Centre, Ahmedabad, IND; 3 Cardiology, University of Pittsburgh Medical Center (UPMC) Harrisburg, Harrisburg, USA

**Keywords:** echocardiography - heart failure - valvular heart disease, heart failure, infective endocarditis, mitral regurgitation, mitral valve aneurysm

## Abstract

Infective endocarditis (IE) is a life-threatening condition caused by infection of the heart’s endocardial surface, often involving native or prosthetic valves. It presents with diverse symptoms, ranging from isolated fever and heart failure to more severe manifestations such as ischemic stroke from septic or thrombus embolization. A rare but serious complication of IE is mitral valve aneurysm (MVA), a localized outpouching of the mitral valve, often leading to mitral regurgitation (MR), heart failure, and, in severe cases, rupture. This report highlights a case of delayed diagnosis of IE in a 34-year-old female who presented with prolonged fever, progressive dyspnea, and signs of heart failure. Echocardiography revealed severe MR, anterior mitral valve leaflet perforation, and a large pseudoaneurysm, suggesting MVA. Because of the advanced presentation, the patient developed refractory heart failure and electrical storm, leading to a fatal outcome before surgery could be performed. This case underscores the importance of early recognition of IE and MVA and the challenges in managing complicated cases, where mitral valve replacement is the preferred surgical option. Early diagnosis and timely intervention are crucial to prevent severe complications and improve prognosis.

## Introduction

Infective endocarditis (IE) is an infection of the heart's endocardial surface, affecting both native valves and prosthetic materials like artificial valves, pacemakers, and catheters. Despite diagnostic and therapeutic advances, IE remains a life-threatening disease with diverse presentation, varying from patients presenting with isolated fevers and decompensated heart failure to patients presenting with hemorrhagic or ischemic stroke resulting from either septic or thrombus embolization [1]. Complications of valvular IE include abscesses, fistulas, thromboembolism, septic embolization, and congestive heart failure from conduction system involvement and valve destruction [2]. Mitral valve aneurysm (MVA) formation was described as early as 1729 by Morand [3]. Transesophageal echocardiography (TEE) is particularly effective in identifying these aneurysms, which appear as saccular outpouchings of the mitral valve leaflets [4]. The integration of echocardiography and CT in the diagnostic workflow enhances the detection and management of IE complications, including MVA formation and rupture, thereby improving patient outcomes [5,6]. Aneurysm rupture in a longstanding MVA can lead to communication with the left atrium (LA), causing mitral regurgitation (MR), and the acute volume overload on the left ventricle (LV) and LA results in pulmonary edema and cardiogenic shock, which can be life-threatening [6,7]. Beyond MR, the rupture can also cause systemic embolization if thrombi are present within the aneurysm, further complicating the clinical picture [4]. Early diagnosis and treatment of IE are crucial to prevent severe complications [8]. We report a case of delayed recognition of IE that resulted in a rare but severe complication of ruptured MVA, causing MR, which resulted in the death of our patient.

## Case presentation

A 34-year-old female patient was admitted to our hospital for prolonged high-grade intermittent fever for three months and worsening breathlessness gradually for two months, from New York Heart Association (NYHA) class II to class IV, along with constitutional symptoms like anorexia, weight loss, and fatigue. No significant past medical history was present, including major cardiovascular conditions. No major risk factors for IE were noted, like IV drug use, cardiac surgery, or congenital heart conditions. Multiple prescriptions of amoxicillin-clavulanic, levofloxacin, and sulfamethoxazole-trimethoprim were given for fever, but the patient never completed the treatment, as per the history. On presentation, her vital signs were as follows: blood pressure (BP) of 100/60 mmHg, heart rate (HR) of 50/minute, respiratory rate (RR) of 18/minute, and oxygen saturation of 95% on room air. Physical examination revealed anemia and mild splenomegaly. Cardiac examination showed a gallop rhythm, a wide split-second heart sound (S2), and a grade III/VI pansystolic murmur at the apex, suggesting severe MR.

**Figure 1:**
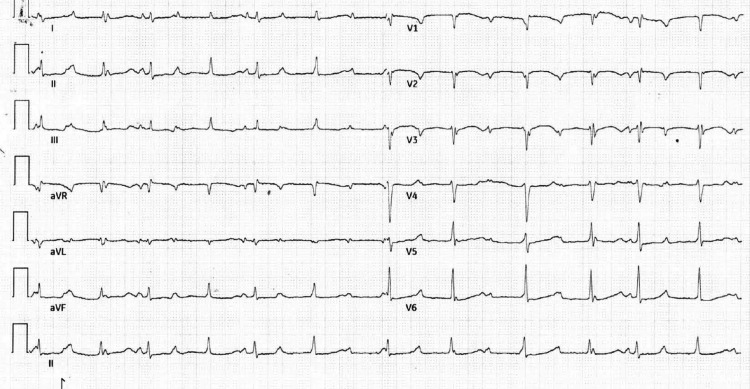
12-lead electrocardiogram (ECG) with junctional rhythm with intermittent complete heart block (CHB)

**Figure 2:**
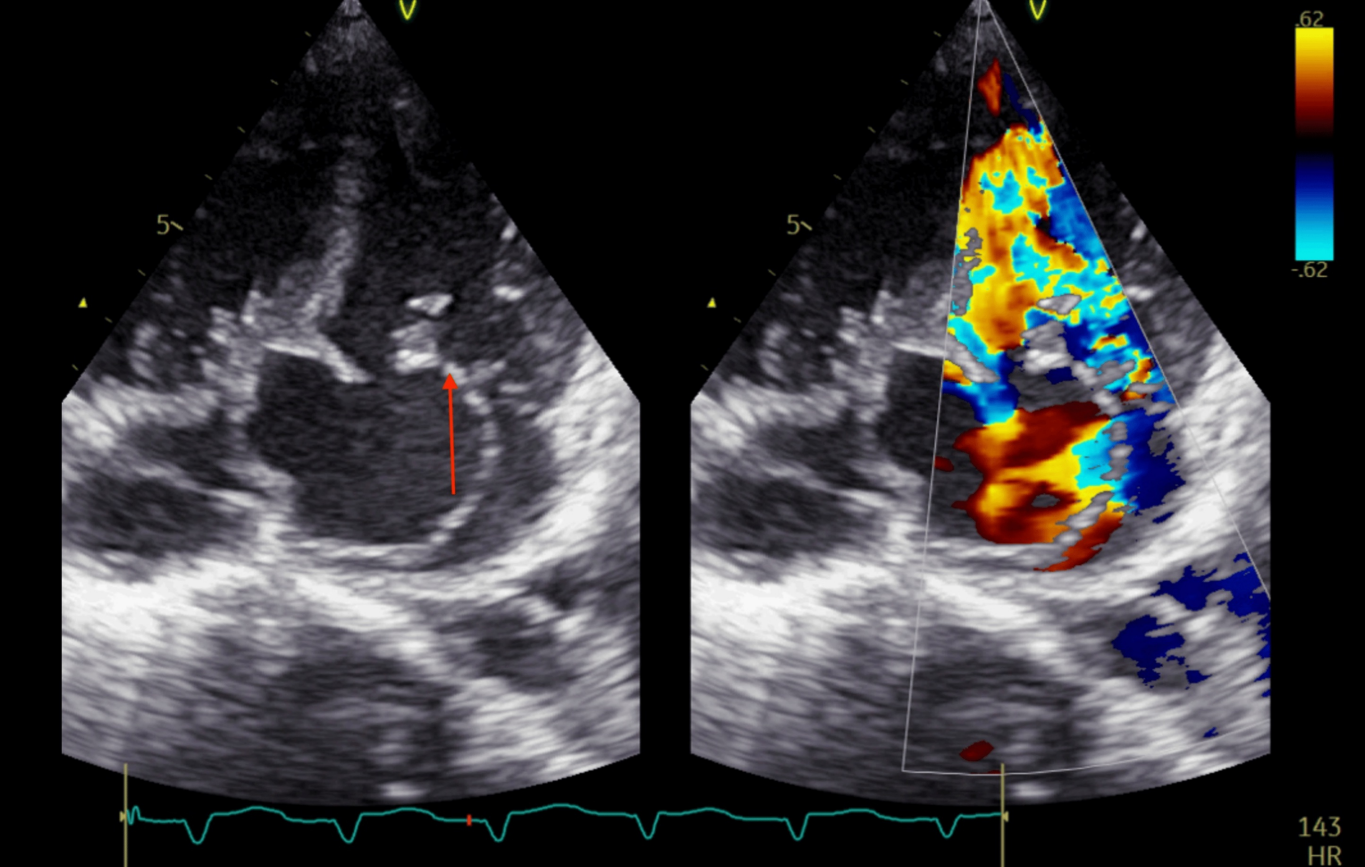
Transthoracic echocardiography showing anterior mitral leaflet perforation (red arrow) with severe mitral regurgitation demonstrated on color Doppler

**Figure 3:**
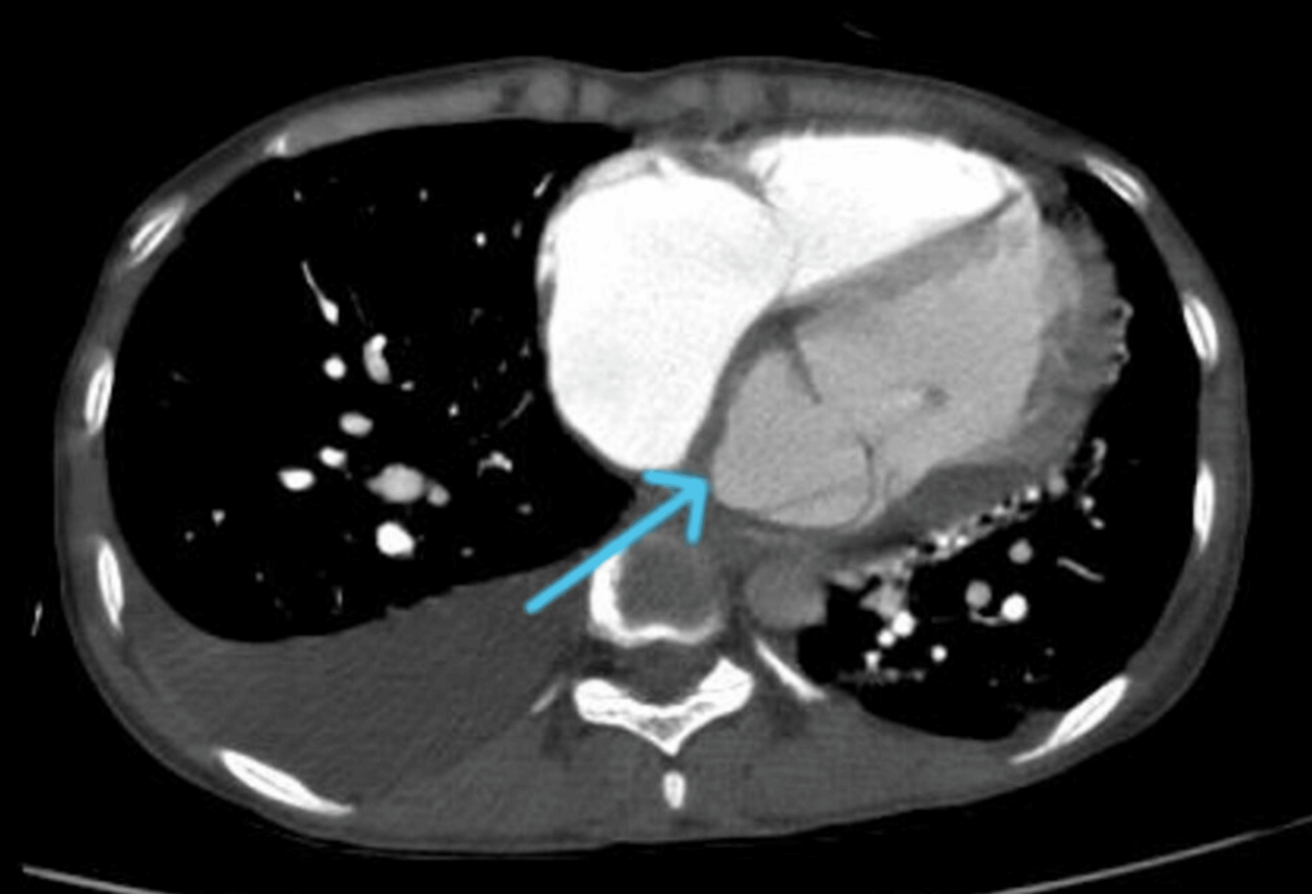
Axial contrast-enhanced cardiac CT image of the chest showing a large 40 × 29 mm pseudo-aneurysm (blue arrow) arising from the basal segments of the left ventricle

A 12-lead electrocardiogram (ECG) revealed a junctional rhythm with intermittent complete heart block (CHB) (Figure 1). Inflammatory markers were elevated, with a total leukocyte count of 20,000/mm³ (80% neutrophils, 18% lymphocytes), an erythrocyte sedimentation rate (ESR) of 80 mm/hour, and a C-reactive protein (CRP) level of 200 mg/L. Additional laboratory values on admission included: hemoglobin 8.5 g/dL, troponin 13 ng/L, pro-BNP 1200 pg/mL, procalcitonin 50 ng/mL, blood urea nitrogen (BUN) 25 mg/dL, creatinine 1.2 mg/dL, sodium 135 mEq/L, potassium 4.5 mEq/L, magnesium 2.2 mg/dL, and phosphorus 3.3 mg/dL (Table 1). Blood cultures were performed but remained negative, likely due to prior antibiotic use before admission. This may have also contributed to the absence of visible vegetation on imaging. A 2D transthoracic echocardiogram (TTE) suggested anterior mitral valve leaflet perforation with severe MR. Additionally, a large subaortic aneurysm arose from the LV and prolapsed into the LA (Figure 2, Video 1). However, there was no evidence of intracardiac vegetation. Cardiac computed tomography revealed a large 40 × 29 mm pseudo-aneurysm arising from the basal segments of LV (Figure 3), involving the aorto-mitral region and projecting into the interatrial septum (IAS) and posterior atrioventricular groove the detailed anatomical information provided by cardiac CT is invaluable for planning the surgical intervention, ensuring that all infected and compromised tissues are addressed, and minimizing the risk of complications during and after surgery. As per the cardiothoracic surgery team, these CT findings may cause significant challenges for surgical planning, as they increase the risk of fistula formation and extensive tissue destruction.

**Table 1:** Laboratory values BUN - blood urea nitrogen; MCH - mean corpuscular hemoglobin; MCHC - mean corpuscular hemoglobin concentration; MCV - mean corpuscular volume; Pro-BNP - pro-brain natriuretic peptide; WBC - white blood cell count

Laboratory parameters	Patient value	Reference range
WBC	20,000/mm^3^	4.30-11.0 mm^3^
Neutrophil	80%	50-65%
Lymphocytes	18%	25-40%
Hemoglobin	8.5 g/dL	14-18 g/dL
MCV	86.2 fL	80-94 fL
MCH	27.3 pg	26-33 pg
MCHC	31.6 g/dL	31-36 g/dL
BUN	25 mg/dL	6-20 mg/dL
Creatinine	1.2 mg/dL	0.7-1.2 mg/dL
Sodium	135 mEq/L	136-145 mEq/L
Potassium	4.5 mEq/L	3.5-5.1 mEq/L
Magnesium	2.2 mg/dL	1.6-2.6 mg/dL
Phosphorus	3.3 mg/dL	2.5-4.5 mg/dL
Troponin	13 ng/L	0-22 ng/L
Pro-BNP	1200 pg/mL	1-125 pg/mL
Procalcitonin	100.2 ng/mL	0.02-0.08 ng/mL

**Video 1:** Transthoracic echocardiogram demonstrating anterior mitral leaflet perforation with severe mitral regurgitation on color Doppler

Treatment

**Figure 4:**
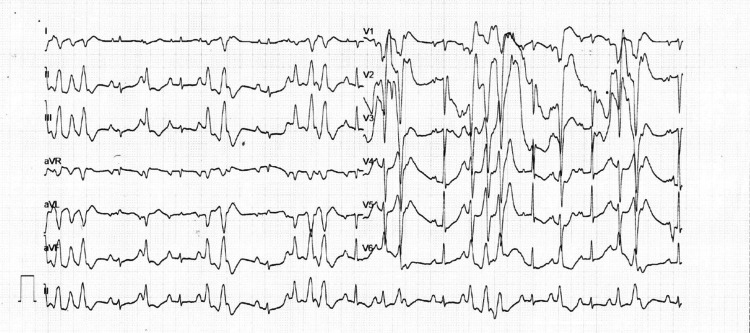
Electrocardiogram showing an electrical storm with recurrent episodes of polymorphic ventricular tachycardia

Considering a clinical suspicion of IE, she was treated with intravenous antibiotics, along with a diuretic and non-invasive ventilation. She was scheduled for early mitral valve replacement (MVR) with pseudoaneurysm repair. However, during the hospital stay, she developed an electrical storm (Figure 4) with multiple episodes of ventricular tachycardia, which was managed with electrical cardioversion, intravenous amiodarone infusion, and overdrive pacing. However, the episodes were only partially responsive, likely due to the underlying severe structural and hemodynamic compromise. Temporary pacing was also attempted to stabilize the HR, but the patient’s condition remained unstable. A multidisciplinary team aimed to stabilize her for definitive surgical intervention. However, she had refractory heart failure precipitated by the electrical storm and succumbed before she could undergo surgical intervention.

## Discussion

MVA is a rare but potentially life-threatening condition that involves the formation of a localized bulge or outpouching in the mitral valve leaflet, with the anterior valve leaflet more commonly involved than the posterior leaflet [9]. The mechanism behind MVA formation is believed to be related to inflammatory processes, causing the valve tissue to weaken and leading to the ballooning of the valve leaflet. This may be induced by endocarditis, rheumatic disease, and other connective tissue diseases like mitral valve prolapse, osteogenesis imperfecta, Marfan syndrome, and pseudoxanthoma elasticum [10]. MVAs present with symptoms of severe MR, including heart failure, dyspnea, and fatigue, similar to the presentation in this case. Auscultatory findings, such as a pansystolic murmur, are typical and reflect the regurgitant flow due to valve dysfunction [11]. The condition often leads to complications such as valvular perforation, rupture, or formation of a pseudoaneurysm, which can severely impair cardiac function and worsen heart failure [12]. Echocardiography is the primary diagnostic tool, revealing the aneurysmal bulge in the mitral leaflet and often showing associated MR [13].

MVAs present significant complications, including the risk of rupture and valvular perforation. While a conservative approach with serial follow-up is often recommended for uncomplicated MVA, when complications such as rupture or severe regurgitation arise, especially in large unruptured aneurysms, surgical intervention becomes necessary [14]. The 2020 American College of Cardiology (ACC)/American Heart Association (AHA) guidelines outline medical and surgical approaches for managing MVAs [6]. Medical management includes antibiotic therapy for MVAs associated with IE to treat the underlying infection and prevent complications. Surgical options depend on the aneurysm's size and valve anatomy. Mitral valve repair is preferred for small aneurysms with favorable anatomy, while MVR is recommended for large unruptured aneurysms, particularly in cases of rupture, severe regurgitation, or when repair durability is uncertain. The guidelines emphasize surgical intervention for symptomatic patients with severe primary MR, irrespective of left ventricular systolic function, and favor MVR in complex cases to ensure durable outcomes. Recent studies support these recommendations, highlighting the importance of surgical management in severe mitral valve pathology [14,15].

In this case, the patient presented with high-grade fever, progressive dyspnea, and signs of heart failure (NYHA classes II to IV), which are typical of severe MR and IE. The echocardiogram showed anterior mitral valve leaflet perforation with severe MR, which is suggestive of an MVA or severe valvular damage. Moreover, the presence of a large pseudoaneurysm arising from the basal segments of the LV further supports the diagnosis of a complex MVA with associated complications, such as pseudoaneurysm formation [14]. The patient's refractory heart failure, despite medical management and antibiotic therapy, is a common outcome in cases of MVA with IE. Surgical intervention, such as MVR and aneurysm repair, is often required, but the presence of an electrical storm and ventricular tachycardia in this patient complicated the clinical course, preventing surgery and leading to a fatal outcome.

## Conclusions

MVAs are a rare but serious complication, often associated with IE, present like MR clinically, and may occur as an isolated pathology. Early diagnosis through echocardiography is crucial. Surgical replacement remains the treatment of choice for patients with severe MR and aneurysm, but the presence of electrical storm and arrhythmias complicates management, as demonstrated in this case.
